# Side population rather than CD133^+ ^cells distinguishes enriched tumorigenicity in hTERT-immortalized primary prostate cancer cells

**DOI:** 10.1186/1476-4598-10-112

**Published:** 2011-09-14

**Authors:** Jianjun Zhou, Honghe Wang, Virginetta Cannon, Karen Marie Wolcott, Hongbin Song, Clayton Yates

**Affiliations:** 1 Department of Biology and Center for Cancer Research, Tuskegee University, Tuskegee, AL 36088 USA; 2 Biomedical Instrumentation Center, Uniformed Services University of the Health Sciences, Bethesda, MD 20814, USA; 3 Institute of Disease Control and Prevention, Academy of Military Medical Science, Beijing, 100071, China

**Keywords:** Cancer Stem Cells, CD133, Side population (SP), prostate cancer

## Abstract

**Background:**

Subpopulations of cancer cells with the capacity of generating solid tumors have been characterized. In various cancer types, including prostate cancer cells, a side population (SP) and CD133-expressing cells have been proposed as containing a population cancer cells with stem-like ability. Therefore the aim of this work was to determine, in prostate cancer cell lines, the frequency and tumorigenic potential of SP and CD133+ cells.

**Results:**

*In vitro *2D colony-forming assay and sphere-forming assay, Flow cytometry analysis and magnetic cell sorting were utilized to sort CD133+, CD133- and Side population (SP) cells. Our findings indicate that CD44 and integrin α-6 are uniformly expressed in the hTERT cell lines; however, CD133 is expressed only in a small population (< 0.1%). FACS-sorted CD133+ and CD133- cells exhibited similar tumorigenicity *in vitro *and *in vivo*. Additionally, for the hTERT cells, SP rather than CD133 expression showed an 8-fold enhanced tumorigenic potential. The data suggest that SP cells, rather than those with CD133 marker, contain the rare population of CSC capable of producing prostate tumors.

**Conclusion:**

Collectively, our data suggest that although CD133 is expressed only in a small population of hTERT-immortalized prostate cancer cells, it is not likely to be associated with stem cells, as CD133- and CD133+ cells exhibited similar tumorigenicity. However, SP isolated cells, appear to be enriched with tumorigenic stem-like cells capable of generating palpable tumors.

## Introduction

Prostate cancer is the most commonly diagnosed malignancy in men. At the time of diagnosis, approximately 50% of men have clinically advanced disease. Although much effort has been directed toward treatment, no therapy has been developed that effectively treats this disease. The problem of treating prostate cancer is a result of the persistence of cancer-initiating progenitor/stem cells that are found in low frequency. A method for identification of cancer stem cells (CSC) in prostate cancer has not been established.

Several populations of cells have been considered as prostate stem cells [1-4]. CD133, in combination with other markers, was originally utilized to isolate hematopoietic stem cells [5,6] as well subpopulations in mammary gland [7], brain [8], colon [9,10], pancreas [11], and liver cells [12]. Although there is no known function for CD133, it is expressed by developing epithelial cells and is rapidly down-regulated upon differentiation [13-16]. CD133 selection has been used to enrich a population of normal prostate epithelial cells capable of forming acinar-like structures as xenografts, and to derive a population of prostate cancer cells with a higher tumorigenic capacity *in vitro *than its negative counterpart [17]. However, use of CD133+ expression for isolation of cancer-initiating progenitor or stem cells is organ-specific and, for prostate cancer, is not directly associated with a subpopulation capable of self-renewal and tumorigenicity [18].

Some cancer cells have, on their cell surface, ATP-binding cassette transporters (ABCG) that pump out the DNA-binding dye, Hoechst 33342 [19]. These cells are resistant to toxic agents and survive longer than cells committed to differentiation. This subset of cells has been characterized as a side population (SP). SP cells are composed of a rare (0.01-5%) and heterogeneous population that varies with tissue type and stage of development [20,21]. SP cells derived from patients and from metastatic cell lines, exhibit enrichment in stem/progenitor cells or CSC/progenitor cells, particularly in cases where the tissue-specific stem cell markers are not established [22]. Numerous cancer models including hematopoitic, pediatric, ovarian, and prostate cancers have investigated the tumorgenic potential of SP cells [23,24].

Since a cell culture model that closely mimics the pathophysiological conditions of primary prostate tumor development is essential to understanding the generation of tumors from CSCs, we have utilized a newly developed panel of hTERT-immortalized primary prostate cancer cell lines, which are similar to non-immortalized primary prostate cancer cells [25]. The hTERT-immortalized lines were generated from primary human tissues representative of most prostate cancer cases [26].

This study focused on the "cancer stem cell hypothesis," which indicates that primary tumors originate from a minor population of cells. With a panel of hTERT immortalized cell lines, CD133 expression and SP were investigated to determine which population of cells is associated with higher tumorigenicity. The results indicate that, although CD133 is expressed only in a small population (< 0.1%) in the hTERT cell lines, CD133+ and CD133- cells exhibited similar tumorigenicity *in vitro *and *in vivo*. Additionally, in our hTERT-immortalized cell lines, SP cells, but not those with CD133 expression, showed an 8-fold higher tumorigenic potential. Thus, SP cells apparently contain the population of CSCs capable of forming prostate tumors.

## Results

### Prostate cancer cells are inefficient at generating spheres *in vitro *and xenograft tumors *in vivo*

**Figure 1:**
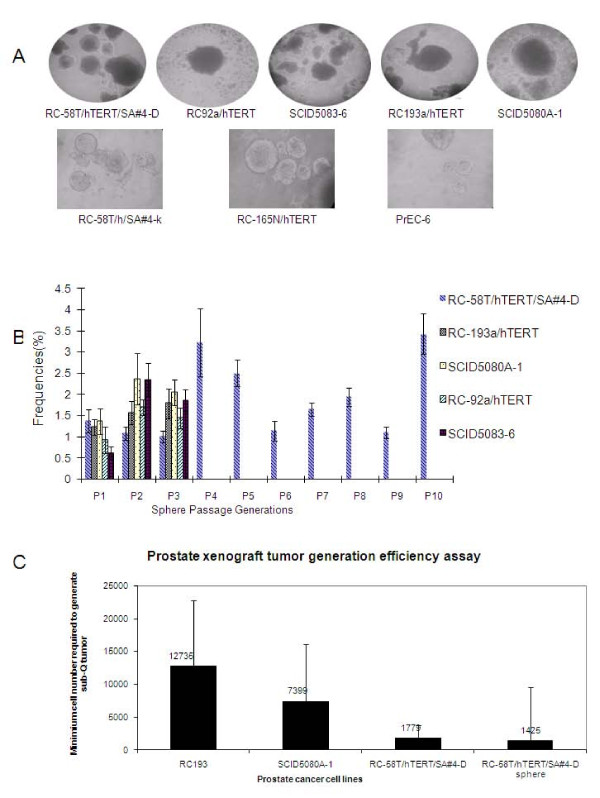
**Tumorigenicity of hTERT-immortalized cells under non-adherent culture conditions and formation of xenograft tumors**. A) hTERT-immortalized epithelial cells lines (RC-58T/hTERT/SA#4-D, RC-92a/hTERT, SCID5083-6, RC-193a/hTERT, and SCID5080A-1, RC-58T/h/SA#4-k, RC-165N/h, PrEC-6) from prostate cancer or non-cancer patients form spheroid structures (called here prostaspheres). The images were taken at 100 × magnification. B) Prostaspheres were serially passaged under non-adherent culture conditions. RC-58T/hTERT/SA#4-D was passaged 10 times without showing a decline in sphere forming capability. The passaging capacities of prostaspheres from RC-92a/hTERT, SCID5083-6, RC-193a/hTERT, and SCID5080A-1 were tested for three generations (n = 6; error bars indicate the standard deviation of sphere-forming efficiency for each type of cell line). C) Xenograft tumor generation with RC193a/hTERT, SCID5080A-1, RC-58T/hTERT/SA#4-D cells cultured as monolayers required large numbers of injected cells. The error bars indicate the lower and upper limits of the confidence interval based on the limiting dilution calculation.

A major characteristic of CSC cells, is their capacity to form three-dimensional structures, or spheres. Thus, we utilized a non-adherent sphere-forming assay to evaluate a panel of hTERT-immortalized human primary prostate cancer cell lines. The assays indicated that the hTERT-immortalized cells contain a minor population, accounting for about 1% of the total, with sphere-forming capacity (Figure 1A). Further, the serial passaging capability of RC-58T/hTERT/SA#4-D spheres was determined. Cells isolated from prostate spheres (prostaspheres) were serially passaged for multiple cycles (Figure 1B), implying that the sphere-initiating cells have self-renewal capability. As controls, non-tumorigenic hTERT-immortalized prostate cell lines RC-58T/h/SA#4-k, RC-165N/hTERT, and PrEC-6 were also tested. Each of these cell lines demonstrated limited serial passaging capabilities (additional file 1).

The rarity of prostasphere formation implied the existence of a minor population of self-renewing cells, we sought to determine if this extended to the generation of tumors in NOD/SCID mice. The limiting dilution assay [27,28] was used to determine the frequency of cells responsible for colony or xenograft generation. In this manner, the tumorigenic potential of RC-193a/hTERT, SCID5080A-1 and RC-58T/hTERT/SA#4-D cells was determined. Generation of palpable tumors required about 12,735 RC193a/hTERT cells; 7,399 SCID5080A-1 cells; 1,799 RC-58T/hTERT/SA#4-D cells cultured in conventional monolayers; or 1,425 RC-58T/hTERT/SA#4-D cells from non-adherent cultured spheres (Figure 1C). Thus, large numbers of cells were required to generate detectable tumors, indicating that prostate tumor growth *in vivo *is an inefficient biological process. RC-58T/hTERT/SA#4D cells generated tumors at significantly lower frequency than RC-193a/hTERT and SCID5080A-1 cells., Non-tumorigenic hTERT-immortalized prostate cell lines RC-58T/h/SA#4-k, RC-165N/hTERT, and PrEC-6 did not give rise to xenograft tumor in mice with comparable large number of cells injection (data not shown).

Since RC-58T/hTERT/SA#4D generated tumors as the lowest frequency, we further sought to determine the expression of several known stem cell markers, Oct 3/4, p63 and ABCG2, before and after *in vivo *inoculation. There was increased Oct 3/4, with similar expression of p63 expression, ABCG2, tumor suppressor, PTEN, and prostate specific cell antigen (PSCA). We did not observe expression of androgen receptor (AR) (additional file 2). In all these findings suggest that cultured cells and xenograft tumors display similar characteristics.

### CD133 does not represent an effective marker for CSCs in hTERT-immortalized prostate cancer cells *in vitro *or *in vivo*

**Figure 2:**
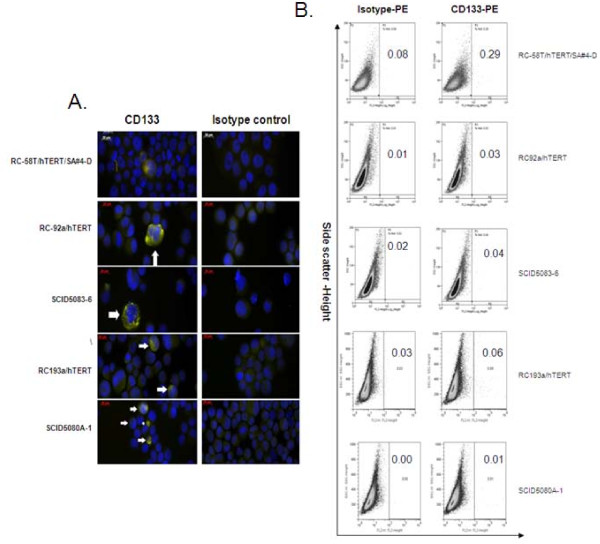
**CD133 phenotyping of hTERT-immortalized prostate cancer cell lines**. RC-58T/hTERT/SA#4-D, RC-92a/hTERT, RC-193/a/hTERT and their xenograft tumor-derived cell lines SCID5083-6, SCID5080A-1 were analyzed for CD133. A). CD133 expression by immunofluorescence utilizing primary CD133 antibody, and Alexa Fluor-594 secondary antibody (yellow). Arrows indicate staining for cell surface molecules. B) CD133 expression by FACS sorting utilizing CD133 antibody conjugated with PE. Indicated within each panel is the percentage of CD133+ cells contained in each cell line.

Additional markers, including CD44, α6 integrin, and CD133, are associated with enrichment of CSCs [29,30]. Since, of these, CD133, the most characterized, is found in enriched fractions of CSCs in several types of cancer tissues (brain, colon, pancreas, and prostate) [17], the expression levels within our panel of hTERT-immortalized prostate cancer cells were determined. As shown by FACS and immunofluorescence analysis of the cell lines, CD133 expression was rare (Figure 2A), amounting to > 0.1% of the cell population (Figure 2B). In contrast, expressions of the cell surface markers, CD44 and α6 integrin, were high and universal (Additional file 3). Thus, CD133 appears to be associated with a minor cell population and could represent the most tumorigenic cells.

**Figure 3:**
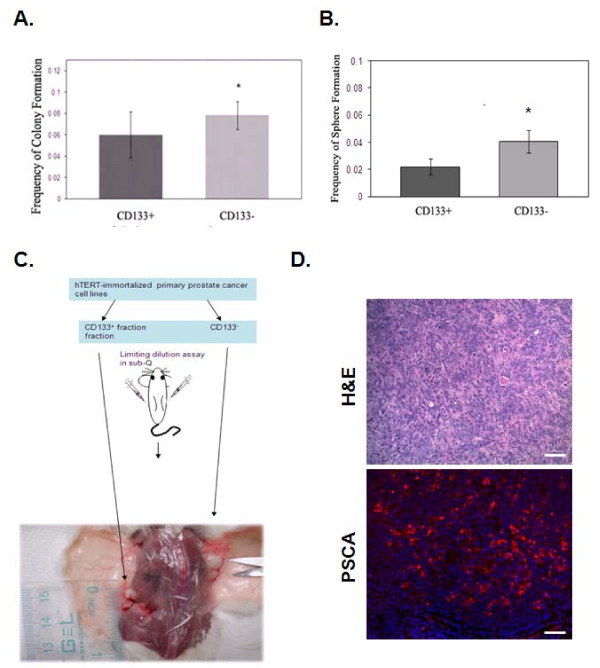
**Lack of enhanced tumorigenicity of CD133+ enriched cells**. A). Double FACS sorted CD133+ subpopulations were assayed for colony-forming advantage relative to the CD133- subpopulation. A) 2D colony-forming assay or B) sphere-forming assay. Data are expressed as means ± standard deviations. Statistical comparisons are with the CD133+ subpopulation, *P = 0.1; **P < 0.05. C). Schematic depiction of assay in mice with CD133-fractioned RC-58T/hTERT/SA#4-D cells as a representative of all tested hTERT-immortalized primary prostate cancer cell lines. In NOD-SCID mice, CD133- cells generated larger tumors than CD133+ cells. D). Formalin-fixed, paraffin-embedded tissues were obtained from xenograft tumors. Individual tissues sections were stained with H&E or human specific primary PSCA antibody, and Alexa Fluor-594 secondary (red) and Dapi (blue) nuclear stain. Scale bar indicates 40 μM.

To determine the role of CD133+ cells versus CD133- cells, double FACS sorting was used to ensure enrichment the CD133+ cells from the RC-58T/hTERT/SA#4-D cell line. CD133+ and CD133- cells were evaluated for formation of two-dimensional (2D) monolayer colonies and for formation of prostaspheres. CD133- cells gave rise to significantly more prostaspheres in monolayer cultures and in non-adherent cultures (P < 0.05) (Figure 3A, B). To characterize further the tumorigenic potential of the CD133+ fraction over CD133- fraction, both were directly inoculated into NOD/SCID mice. CD133- cells displayed higher tumorigenicity (Figure 3C). Quantitatively, CD133+ fractions have a 1/28,854 frequency of generating tumors compared to a 1/3,002 frequency for CD133- fractions in RC-58T/hTERT/SA#4-D (Figure 3C and Table 1). This trend was confirmed, under similar conditions, for fractions in RC-193/hTERT (CD133+, < 1/24,836 and CD133-, 1/14,244) and SCID5080A-1 (CD133+, 1/5,586 and CD133-, 1/1468). To confirm that these tumors where of human and prostate origin we performed H&E staining and immunofluorescence staining utilizing human specific prostate specific stem cell antigen (PSCA) antibody. PSCA expression was significantly expressed in xenograft tumor cells and staining was not observed in the cells of surrounding stroma (Figure 3D). Additionally, secondary antibody alone did not reveal any positive staining (data not shown). Thus, it appears that, in this model system, CD133 expression is not sufficient to determine the tumorigenic potential of prostate cancer cells.

**Table 1:** Tumorigenicity of CD133+ or CD133- Fractions in hTERT immortalized cell lines.

*RC-58T/hTERT/SA#4-D*			
Cell Types	Cell number	Incidence	Estimated frequency
**CD133+** (n = 23)	100	0/3	1/28,854 (1/3,908 ~ 1/213,084)
	500	0/3	
	1,000	0/4	
	2,000	0/4	
	5,000	0/3	
	10,000	0/4	
	20,000	1/2	
**CD133-** (n = 23)	100	0/3	1/3,002 (1/1,408 ~ 1/6,401)
	500	0/3	
	1,000	2/4	
	2,000	4/4	
	5,000	2/3	
	10,000	3/4	
	20,000	2/2	
***RC-193a/hTERT***			
**CD133+** (n = 16)	100	0/4	< 1/24,836
	500	0/4	
	1,000	0/4	
	10,000	0/2	
	24,000	0/2	
**CD133-** (n = 24)	100	0/4	1/14,244 (1/5,880~1/34,508)
	500	0/4	
	1,000	0/8	
	10,000	3/6	
	24,000	2/2	
***SCID5080A-1***			
**CD133+** (n = 19)	100	1/4	1/5,586 (1/1,901~1/16,419)
	500	1/3	
	1,000	1/4	
	2,000	2/4	
	18,000	1/2	
	30,000	2/2	
**CD133-** (n = 19)	100	2/4	1/1,468 (1/614~1/3,365)
	500	2/3	
	1,000	1/4	
	2,000	2/4	
	18,000	2/2	
	30,000	2/2	

For hTERT-immortalized human primary prostate cancer cell line RC-58T/hTERT/SA#4-D, NOD/SCID mice were killed at 16 weeks post-injection. For RC-92a/hTERT, mice were killed at 23 weeks post-injection since RC-92a/hTERT xenograft tumor grows very slowly. For SCID5083-6, mice were killed at 16-17 weeks post-injection. For RC-193a/hTERT, mice were killed at 15-23 weeks post-injection. For SCID5080A-1, mice were sacrificed at 15-23 weeks post-injection. Mice were considered positive if palpable tumor tissue was identified. All the doses except that only resulted in negative mice were used to calculate the limiting dilution experiments. Confidence interval was displayed in the bracket. SCID5083-6 and SCID5080A-1 xenograft tumors grows faster than its parental RC-92a/hTERT, RC-193a/hTERT xenograft tumors, respectively.

### SP isolated cells have higher tumorigenicity than CD133+ cells in hTERT immortalized prostate cancer cells

**Figure 4:**
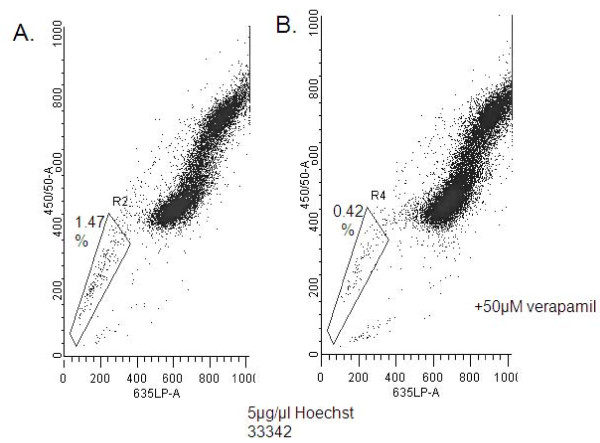
**SP in RC-58T/hTERT/SA#4-D cells**. A) RC-58T/hTERT/SA#4-D cells were analyzed for SP based on Hoechst 33342 (Sigma) or DCV staining. The RC-58T/hTERT/SA#4-D SP cells represent 1.47% of the entire population. B) 0.42% of the RC-58T/hTERT/SA#4-D SP as inhibited by 50 μM verapamil.

**Figure 5:**
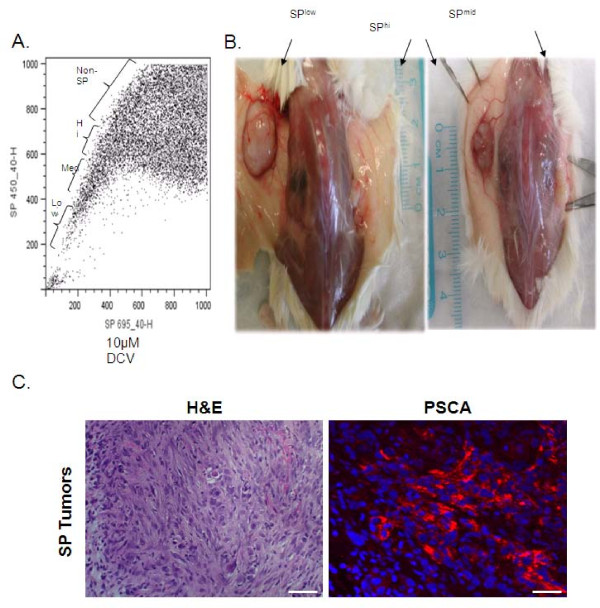
**Analysis of SP and non-SP used for cell isolation and for animal experiments**. A) RC-58T/hTERT/SA#4-D cells were subjected to Hoechst 33342 dye and separated into SP^low^, SP^mid^, and SP^hi ^fractions B). Individual SP fractions were then injected subcutaneously and assayed for tumorigenicity, after 100 days in NOD-SCID mice. Tumors were subsequently measured for tumor volume as shown by schematic representing xenograft tumors. D). Formalin-fixed, paraffin-embedded tissues were obtained from SP generated xenograft tumors. Individual tissues sections were stained with H&E or human specific primary PSCA antibody, and Alexa Fluor-594 secondary (red) and Dapi (blue) nuclear stain. Scale bar indicates 40 μM.

Since CD133 expression failed to determine tumorigenicity, we asked if there existed a minor cell population, representing the CSCs in the hTERT cells. Since SP analysis has been used to identify stem cell populations [2,31-33], we examined the SP of RC-58T/hTERT/SA#4-D cells, since this line gave rise to subcutaneous xenograft tumors in NOD/SCID mice in a shorter time relative to other hTERT-immortalized cell lines. Utilizing 5 μg/ml of Hoechst 33342 or DyeCycle Violet (DCV), we determined that 1.47% of the RC-58T/hTERT/SA#4-D cell population was positive and verapamil-sensitive (Figure 4A, B). Xenograft tumor growth indicated that SP-isolated cells showed increased tumorigenic frequency (6~8 fold) over non-SP cells (Table 2). SP cells also formed larger xenograft tumors in a shorter time than non-SP cells. The SP "arm" was divided into three portions, SP^low^, SP^mid^, and SP^hi ^(Figure 5A), to determine the tumorigenicity of each population of cells. This fractionation into sub-groups did not yield a significant tumorigenic enhancement, i.e., SP^low ^(7.37-fold), SP^mid ^(6.55-fold), and SP^hi ^(7.98-fold), over non-SP cells, although the tumors generated by SP^low ^were larger than those generated by other SP fractions (Figure 5B). Additionally, in some cases, non-SP cells gave rise to small xenograft tumors (data not shown). Immunofluorescence staining for human specific PSCA expression, confirmed tumors were human and of prostate origin as well (Figure 5C).

**Table 2:** In vivo Tumorigenicity of Side Population Isolated cells.

*RC-58T/hTERT/SA#4-D*			
Cell Types	Cell number	Incidence	Combined Estimated frequency
SP^low^	1,000	2/5	1/3,522
^(n = 18)^	5,000	2/4	(1/1,661~1/7,466)
	10,000	6/6	
	50,000	3/3	
SP^mid^	1,000	1/5	1/3,963
^(n = 15)^	5,000	4/4	(1/1,762 ~1/8,914)
	10,000	3/4	
	50,000	2/2	
SP^hi^	1,000	2/4	1/3,252
^(n = 14)^	5,000	4/4	(1/1,356~1/7,805)
	10,000	2/3	
	50,000	2/2	
Non-	1,000	1/4	1/25,952
SP	5,000	3/4	(1/9,762~1/68,998)
^(n = 14)^	10,000	1/3	
	50,000	1/3	

** Mice were killed at 15-17 weeks post-injection. All the cell doses, excluding negative mice, were used to calculate the limiting dilution experiments. 95% confidence interval was displayed in the bracket. Combined Frequency for SP = 1/3,574 (1/2,243~1/5,695)

Since SP enriched population isolated cells indicated tumorigenicity, we further sought to characterize CD133 expression in the non-SP and SP cells. Interestingly, 2.3% of cells were CD133+ in non-SP sorted cells, which is similar to findings in Figure 2, however there was only 1.5% of cells that were CD133+ in the DCV sorted SP cells (Additional file 4). The results indicate that, while SP isolation provides enrichment of the CSC population. Further characterization of additional markers associated with SP is warranted.

## Discussion

A small subset of cancer cells may be responsible for tumor development and recurrence after therapy. In prostate cancer, there appears to be a subset of cancer cells, with properties of adult stem cells [1], that can self-renew and generate differentiated cancer cells with limited proliferative potential. In addition, CSCs, which are rare, are considered to possess chemoresistance and teleomerase activity and to have specific gene signatures and signal pathway activities [34]. Since cancers are heterogeneous and all cells do not behave in the same manner, the cancer stem cell theory provides a plausible explanation to these events. CSCs or stem-like cancer cells originate from a small fraction of cells. They have the capacities of self-renewal, and chemoresistance, and they give rise to large numbers of cells within the tumor mass [35]. The hypothesis that the tumors originate from a minor cell subset with stem cell characteristics has been demonstrated by studies with breast, brain, prostate, and other solid tumors [4,7,8,11]. Furthermore, CSCs have been suggested to be responsible for tumorigenicity, progression, and metastasis in cancers [36]. Therefore, identification and characterization of this population of cells is needed to understand their characteristics and to develop treatment strategies targeting this small population [37].

Efforts have been made to determine the physical markers associated with CSCs [38]. For prostate cancer, there has been difficulty in harvesting quality samples of tissues and in generating cell lines for research purposes. LNCaP, DU145, PC3 (PPC1), and 22Rv1 are most popularly as cell culture models. These cells are all isolated from metastatic patients and thus are not useful for determining development of primary tumors. To determine the CSC population capable of generating tumors, we utilized a panel of hTERT immortalized cell lines. Initially, we determined that all hTERT immortalized cells have sphere-forming capacity and that they can be serially passaged. These findings are similar to those for previously established, immortalized hTERT prostate cells [39] and for prostate clinical samples [40]. Since, to date, there have been no reports of how this translates into tumor growth *in vivo*, bulk population cells or only sphere-forming cells were inoculated into NOD/SCID mice. Among these populations, there was no difference in tumor formation (Table 1). Molecular characterization of the protospheres before and after in vivo inoculation showed similar expression levels of CD44, Oct 3/4, p63. Further characterization of prostate specific markers showed AR expression is absent, however there was robust expression of PSCA. Several reports have identified these markers, including the lack of AR expression to be associated with prostate cancer stem cells [41] (Supplemental Figure 1), which further suggest that sphere-forming capacity or generally utilized molecular makers typically associated with minor cell population, do not completely characterize the CSC population. Furthermore, our use of multiple cell lines decreases the possibility of distinct cell line characteristic as an explanation of these results.

Consistent with the stem cell hypothesis, which indicates that only a minor fraction of cells are capable of generating tumors, CD133 expression was observed, by immunofluorescence and FACS, only in a minor fraction of cells, > 1% of the population. As determined with FACS-sorted CD133^+ ^and CD133 ^-^RC-58T/hTERT/SA#4-D cells, CD133 expression did not have an influence on the capacity of cells to form prostaspheres or on tumor development in SCID mice. In fact, CD133- cells formed larger prostaspheres and larger tumors in mice. Similarly, Shmelkov and colleagues [42] reported that, for a mouse model of colon cancer, the CD133^+ ^and CD133^- ^tumor subpopulations formed colonospheres *in vitro *and were tumorigenic in a mouse model involving NOD/SCID serial xenotransplantation, with CD133^- ^cells forming more aggressive tumors [42]. The present results and those by Shmelkov et al. are contradictory to reports from Miki [29] and Goodyear [39], that hTERT-immortalized prostate cells containing a CD133^hi ^subpopulation (< 3.3%), in contrast to CD133^lo ^cells, generated prostaspheres *in vitro *and dysplastic lesions in NOD-SCID mice [39]. Interestingly, however only cells from CD133^hi ^prostaspheres, but not CD133^hi ^single cells, were capable of generating dysplastic lesions. Since CD133^+ ^cells have been reported to give rise to CD133^- ^cells [8,10], suggest that additional CD133^lo ^cells are required to support growth *in vivo *and that CD133 cells alone are not capable of generating tumor development.

Since CD133 expression did not determine the tumorigenic potential of RC-58T/hTERT/SA#4-D cells, we utilized SPs, which allows isolation of a small population of cells that differentially pump out the fluorescent dye, Hoechst 33342, by the pump proteins, ABC transporters, on the cell surface [43], as a second method to determine the minor population capable of developing tumors in mice. For RC-58T/hTERT/SA#4-D cells, the results suggested successfully isolation of minor populations of cells, SP^low^, SP^mid^, and SP^hi ^(Figure 5). This was confirmed with displacement with 50 μM verapamil (Figure 4A). After inoculation of SP^low^, SP^mid ^and SP^hi ^populations of isolated cells into NOD/SCID, SP^low^-enriched prostate cancer cells developed tumors (Table 2). Although SP^mid ^and SP^hi ^isolated cells also developed tumors, SP^low ^cells developed tumors at a faster rate, and tumor size was significantly larger (Table 2, Figure 5). This suggested that SP, in contrast to CD133 expression is useful in determining the population of cells capable of giving rise to tumors.

Our results show that tumors at different clinical stages are heterogeneous. Nevertheless, CSC identified in cultured cancer cells could be identified in intact animals. Furthermore, our findings suggest that SP is a more appropriate method for determining the tumorigenic potential of small populations of cells, as both SP and non-SP cells express a similar percent of CD133+ expressing cells. Thus further characterization of this SP population is warranted. SP cells have been identified in several tumor cell lines [33,44,45] as well as in fresh tumor samples [46-48]. There are, however, few reports of their presence in prostate cancer. However, work with prostate and breast cancer cells shows that ABCG2-positive and -negative cells have similar tumorigenicity [2]. Further, a recent report concerning DU-145 and PC-3 cells showed that SP cells contain a large CD133+ population [22]. Although there was no characterization of the tumorigenicity of this population, the results highlight the fact that further characterization of the SP with physical markers such as CD133 will aid in the discovery of tumor-initiating CSCs.

Apparently, numerous factors determine the tumorigenic potential of putative stem cells, and xenografts tumor models are essential for complete characterization of the tumor-initiating CSC. Identification of a subset of CD133-expressing CSCs from human cancer samples may not be ideal. Contributing to tumor growth in animals are various factors, such as the tumor microenvironment and transplantation site. These should be well controlled, and results should be cautiously interpreted considering the physiological conditions of organ-specific development of tumors. Studies with multiple cancer types, including melanomas, suggesting an association between CSC frequency and a particular marker with tumorigenic potential should be reassessed with optimized experimental conditions, especially in a assay involving intact animals [49,50]. It is essential to identify the physical markers and experimental conditions that initiate tumors in patients.

## Conclusion

Prostate cancer is difficult to treat, for its stem cells are poorly defined and therefore are untargeted. This report presents evidence, that through quantitative assays in intact animals, that SP is responsible for the population of cells necessary for tumor development.

## Materials and methods

### Cell samples and culture

RC-58T/hTERT/SA#4-D, RC-58T/h/SA#4-k [51], RC-92a/hTERT [29], RC-193-a/hTERT, RC-165N/hTERT [52], and PrEC-6 [53] cell lines were generated by hTERT-immortalization of primary human prostate cancer or benign tissues from different patients. SCID5083-6 and SCID5080A-1 are cell lines from xenograft tumors induced by RC-92a/hTERT cells [29] and RC-193a/hTERT cells, respectively. The basic procedures for establishing and characterizing prostate cell models have been described [54]. Passage number for individual fractions of cells are indicated as follows: RC58T/h/SA#4(D) SP and Non-SP p17 RC58T/h/SA#4(D) p17 CD133- RC58T/h/SA#4(D) p17 CD133+ RC193 p2 CD133+ RC193 p2 CD133- SCID5080A-1 p8 CD133- SCID5080A-1 p8 CD133+

Most cells lines were cultured in serum-free keratinocyte medium (Invitrogen) and only RC-58T/hTERT/SA#4-D were cultured with DMEM plus 10% serum and insulin (2 μg/ml, Sigma).

### *In vitro *2D colony-forming assay

Cells, plated at a density of 300 cells/ml in 12-well tissue culture plates (Corning), were fixed with 4% paraformaldehyde (PFA) for 20 min at room temperature and stained with a mixture of 1% Nile blue and 1% rhodamine B (in water). Colonies larger than 1 mm in diameter were counted.

### Sphere-forming assay

Non-adherent cultures were conducted as described [55]. Briefly, cells were plated in 6-well, ultra-low attachment plates (Corning) at a density of 1000 cells/ml. After 2-3 weeks, prostaspheres were collected by centrifugation at 800 rpm and dissociated in 0.05% trypsin, 0.53 mM EDTA (Invitrogen) for 10 min and by use of a plastic pipette to disrupt the spheres into single cells mechanically. They were then passed through a 40-μm mesh filter (BD Biosciences). The frequency of sphere-forming were calculated by the number of observed spheres divided by the initial number of seeding cells.

### SP and cell sorting

The SP analysis based on Hoechst 33342 (Sigma) or DCV (Invitrogen) staining was performed as previously described [2,56,57]. For instruments equipped with violet laser, DCV was substituted for Hoechst 33342 SP, since it has emission characteristics and cell permeability similar to Hoechst 33342, but with longer wavelength excitation maxima. Briefly, cells were collected with Accutase (Millipore) from culture flasks and re-suspended in warm culture medium (10^6 ^cells/ml). Hoechst 33342 was added at a final concentration of 10 μg/mL, and cells were incubated at 37°C for 2 h with intermittent mixing. For DCV staining, DCV (10 μM final concentration) was added to cultures, and the cells were incubated for 30 min. As control reactions, verapamil (Sigma) was added at a final concentration of 50 μg/ml in the presence of Hoechst 33342. After incubation, cells were washed once and re-suspended in ice-cold Hanks' balanced saline solution (Invitrogen) containing 2% fetal bovine serum (Invitrogen) and 2 mM HEPES buffer (Invitrogen). Cells were stained with 7-aminoactinomycin D (7-AAD, 2 μg/ml, Sigma) to determine viability, then analyzed and sorted by flow cytometry using FACS Vantage DiVa (Becton Dickinson), FACS Vantage SE or Accuri Flow cytometer.

### Flow cytometry analysis and magnetic cell sorting

Routine flow cytometry analysis was performed with FACS Calibur and a BD LSR II flow cytometer (BD Biosciences). CD44 (Millipore), α6 integrin (R&D), and CD133 antibody conjugated with phycoerythrin (PE) or anti-allophycocyanin (Miltenyi Biotec) were used. For the population analyses, at least 100,000 events were acquired for each sample, and all cells positive for 7-AAD were gated out. For the magnetic cell sorting, RC-58T/hTERT/SA#4-D cells were collected by Accutase (Millipore) digestion and magnetically labeled and separated by double passage using a CD133 Cell direct or indirect isolation Kit (Miltenyi Biotec) according to manufacturer's instruction. CD133+ cells constituted 15.98% of the magnetically sorted viable cells as determined by flow cytometry and where utilized directly without sub-passaging for assays *in vitro *and *in vivo*.

### Immunostaining

Indirect immunostaining was used to detect expression of the cell surface marker. Briefly, cytospined cells were fixed with 4% PFA in phosphate-buffered saline (PBS) for 10 min at room temperature and, after washing with PBS, blocked with 10% goat whole serum in PBS. After washing, cells were incubated sequentially with monoclonal antibody anti-CD133 (Miltenyi Biotec), Alexa Fluor-594, or 546-conjugated goat-anti-mouse secondary antibody (Invitrogen), and 4',6-diamidino-2-phenylindole (Sigma), with washings after each step. They were then placed in mounting medium (Vector). Images were acquired and analyzed with the Zeiss Axio fluorescence imaging system (Carl Zeiss MicroImaging Inc.) in the Tuskegee University Imaging Facility.

### Immunohistochemistry

PSCA staining in prostate xenografts in mice was analyzed in formalin-fixed, paraffin-embedded tissues. Individual tissue sections were then incubated with Hematoxylin and Eosin (H&E) stain or blocked with 3% BSA, 5% NGS, PBS 1-2 hrs. Polyclonal antibody against human specific PSCA (H-83; SCBT) at 1:50 dilution in 3%BSA, 5% NGS, PBST was then applied at RT for 1 h. Secondary anti-rabbit Alexa Fluor-594 (red) was utilized at 1:100 for 1 h, and a counterstain Dapi (blue) was applied.

### Assay of tumorigenicity in intact animals and limiting dilution analysis

NOD/SCID mice were purchased from the Animal Production Area of the National Cancer Institute-Frederick Cancer Research and Development Center (Frederick, Maryland) and maintained in a barrier facility approved by the American Association for Accreditation of Laboratory Animal Care. For injection of cells, a previously published method [2,58] was used. Briefly, 100 μl of cells in their regular culture medium and 100 μl of Matrigel (BD Biosciences) were mixed and injected subcutaneously into mice (4-8 weeks old). All mice were euthanized when the tumor measured 2 cm or between 15 and 26 weeks post-transplantation or when their health ice was threatened. Calculations were based on the limdil function in the statmod package, which is part of R statistical package. The process was executed online http://bioinf.wehi.edu.au/software/limdil/index.html.

### Statistical analysis

Group differences were determined by unpaired Student's t-test. P values of < 0.05 were considered significant.

## Competing interests

The authors declare that they have no competing interests.

## Authors' contributions

JZ conceived, designed, and performed the experiments and analyzed the data. KW, HW, VC performed the major SP analyses and cell sorting by FACS. JZ, HS, and CY wrote the manuscript. All authors have read and approve the manuscript in its final form.

## Supplementary Material

Additional file 1 **Non-tumorigenic hTERT-immortalized prostate cell line show limited serial passaging**. Table summarizes the serial passaging threshold for RC-58T/h/SA#4-k, RC-165N/hTERT, and PrEC-6 cells, respectively. Click here for file

Additional file 2 **Specific Markers for RC-58T/hTERT/SA#4-D**. Analysis of RT-PCR products for Oct 3/4, ABCG2, PTEN, PSCA, and AR were generated from RC-58T/hTERT/SA#4-D cells and corresponding xenograft derived tumors. GAPDH served as internal control. Figure shown is representative data from experiments formed in triplicate, and multiple xenograft tumors. Click here for file

Additional file 3 **CD44 and Integrin α6 phenotyping of hTERT-immortalized prostate cancer cell lines**. RC-58T/hTERT/SA#4-D, RC-193/a/hTERT and their xenograft tumor-derived cell lines SCID5083-6, SCID5080A-1 were analyzed for CD44 and integrin α6. A). CD44 expression was determined by immunofluorescence utilizing primary CD44 antibody, and Alexa Fluor-594 secondary antibody (red). Arrows indicate staining for cell surface molecules. B) CD44 and integrin α6 expression was determined by FACS sorting utilizing CD44 or integrin α6 antibody conjugated with PE, respectively. Click here for file

Additional file 4 **Percentage of CD133 expressing cells in the Side Population and non-Side Population FACS sorted cells**. RC-58T/hTERT/SA#4-D cells were analyzed for CD133 expression in SP or non-SP sorted cell populations. A.) The percentage of the non-side population (SP) cells expressing CD133 was determined utilizing CD133 PE-conjugated antibody. B.) The percentage of DCV FACS sorted (SP) cells expressing CD133 was determined utilizing CD133 PE-conjugated antibody. Indicated within each panel is the percentage of CD133+ cells. C.) Table summarizing the percentage of cells in the non-SP and SP cell fractions that express CD133 positivity. Click here for file
