# Supplementary material for: Side population rather than CD133+ cells distinguishes enriched tumorigenicity in hTERT-immortalized primary prostate cancer cells
Source: Mol Cancer. 2011 Sep 14;10:112. doi: 10.1186/1476-4598-10-112 (PMC3180433; doi:10.1186/1476-4598-10-112)
Supplement: Additional file 1 — Non-tumorigenic hTERT-immortalized prostate cell line show limited serial passaging. Table summarizes the serial passaging threshold for RC-58T/h/SA#4-k, RC-165N/hTERT, and PrEC-6 cells, respectively. [file 1476-4598-10-112-S1.DOC]

| Cell types | Serial passage threshold number |
| --- | --- |
| PrEC-6 | 7 |
| RC-58T/h/SA#4-k | 9 |
| RC-165N/hTERT | 13 |
