# Supplementary figures and images for: Side population rather than CD133+ cells distinguishes enriched tumorigenicity in hTERT-immortalized primary prostate cancer cells
Source: Mol Cancer. 2011 Sep 14;10:112. doi: 10.1186/1476-4598-10-112 (PMC3180433; doi:10.1186/1476-4598-10-112)

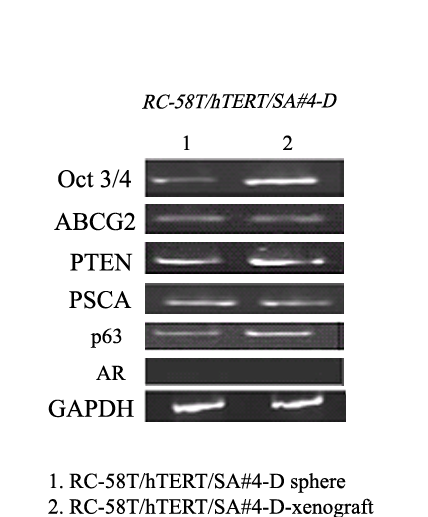

Supplement: Additional file 2 — Specific Markers for RC-58T/hTERT/SA#4-D. Analysis of RT-PCR products for Oct 3/4, ABCG2, PTEN, PSCA, and AR were generated from RC-58T/hTERT/SA#4-D cells and corresponding xenograft derived tumors. GAPDH served as internal control. Figure shown is representative data from experiments formed in triplicate, and multiple xenograft tumors. [file 1476-4598-10-112-S2.TIFF]

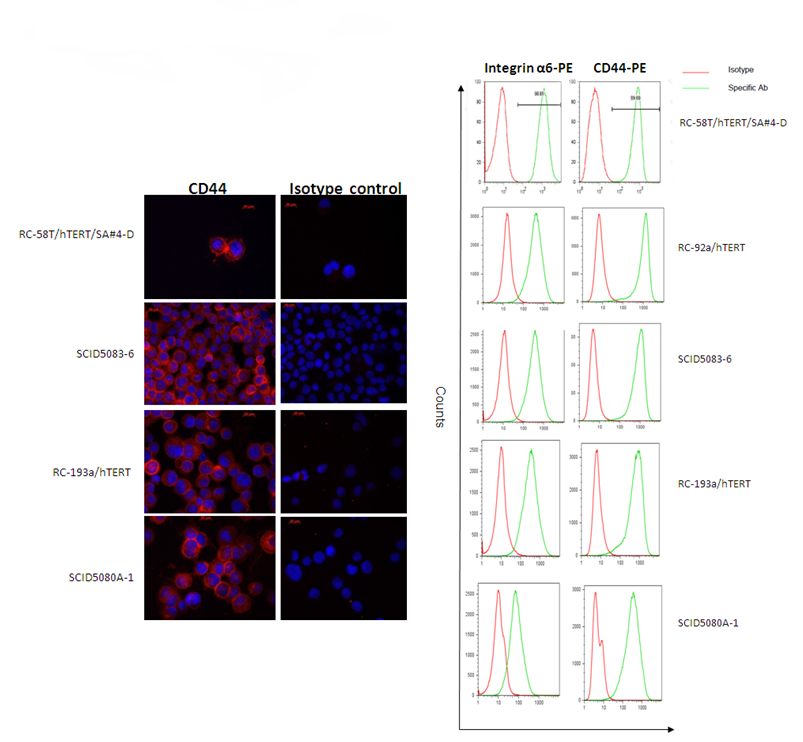

Supplement: Additional file 3 — CD44 and Integrin α6 phenotyping of hTERT-immortalized prostate cancer cell lines. RC-58T/hTERT/SA#4-D, RC-193/a/hTERT and their xenograft tumor-derived cell lines SCID5083-6, SCID5080A-1 were analyzed for CD44 and integrin α6. A). CD44 expression was determined by immunofluorescence utilizing primary CD44 antibody, and Alexa Fluor-594 secondary antibody (red). Arrows indicate staining for cell surface molecules. B) CD44 and integrin α6 expression was determined by FACS sorting utilizing CD44 or integrin α6 antibody conjugated with PE, respectively. [file 1476-4598-10-112-S3.TIFF]

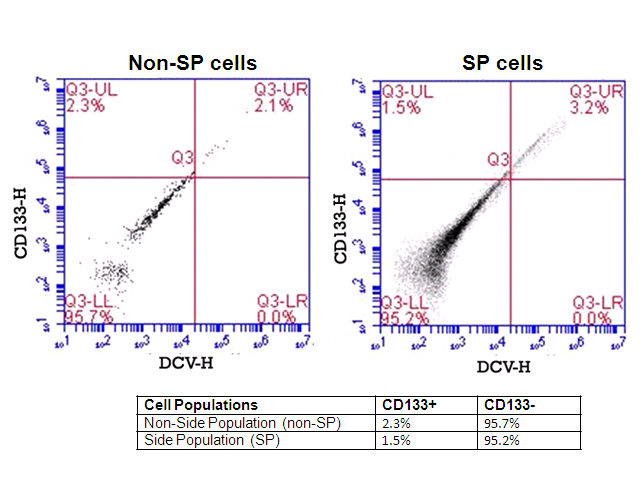

Supplement: Additional file 4 — Percentage of CD133 expressing cells in the Side Population and non-Side Population FACS sorted cells. RC-58T/hTERT/SA#4-D cells were analyzed for CD133 expression in SP or non-SP sorted cell populations. A.) The percentage of the non-side population (SP) cells expressing CD133 was determined utilizing CD133 PE-conjugated antibody. B.) The percentage of DCV FACS sorted (SP) cells expressing CD133 was determined utilizing CD133 PE-conjugated antibody. Indicated within each panel is the percentage of CD133+ cells. C.) Table summarizing the percentage of cells in the non-SP and SP cell fractions that express CD133 positivity. [file 1476-4598-10-112-S4.TIFF]
